# High Resolution Mass Spectrometry of Polyfluorinated Polyether-Based Formulation

**DOI:** 10.1007/s13361-015-1269-9

**Published:** 2015-10-30

**Authors:** Ian Ken Dimzon, Xenia Trier, Tobias Frömel, Rick Helmus, Thomas P. Knepper, Pim de Voogt

**Affiliations:** Institute for Biodiversity and Ecosystem Dynamics, University of Amsterdam, Science Park 904, Amsterdam, 1098XH The Netherlands; Hochschule Fresenius, Institute for Analytical Research, Limburger St. 2, D-65510 Idstein, Germany; The National Food Institute, Mørkhøj Bygade 19, DK-2860 Søborg, Denmark

**Keywords:** HRMS, PFPE, Higher-order mass defect

## Abstract

High resolution mass spectrometry (HRMS) was successfully applied to elucidate the structure of a polyfluorinated polyether (PFPE)-based formulation. The mass spectrum generated from direct injection into the MS was examined by identifying the different repeating units manually and with the aid of an instrument data processor. Highly accurate mass spectral data enabled the calculation of higher-order mass defects. The different plots of MW and the nth-order mass defects (up to n = 3) could aid in assessing the structure of the different repeating units and estimating their absolute and relative number per molecule. The three major repeating units were -C_2_H_4_O-, -C_2_F_4_O-, and -CF_2_O-. Tandem MS was used to identify the end groups that appeared to be phosphates, as well as the possible distribution of the repeating units. Reversed-phase HPLC separated of the polymer molecules on the basis of number of nonpolar repeating units. The elucidated structure resembles the structure in the published manufacturer technical data. This analytical approach to the characterization of a PFPE-based formulation can serve as a guide in analyzing not just other PFPE-based formulations but also other fluorinated and non-fluorinated polymers. The information from MS is essential in studying the physico-chemical properties of PFPEs and can help in assessing the risks they pose to the environment and to human health.

**Electronic supplementary material:**

The online version of this article (doi:10.1007/s13361-015-1269-9) contains supplementary material, which is available to authorized users.

**Graphical Abstract:**
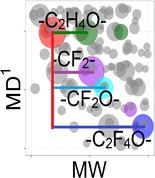
ᅟ

## Introduction

Perfluoropolyalkylether (PFPE) represents a very diverse group of enduring polymers with unique applications. Generally, PFPEs have high thermal and chemical stabilities and low surface energies, dielectric constants, and vapor pressures [1, 2]. Consequently, PFPEs provide low wettabilities and coefficients of friction [3]. As such, these polymers have found a variety of uses as high performance lubricating oils and greases. The most important applications include lubricants for food processing equipment and packaging [4–6]. PFPEs are listed as materials that can be used in materials intended for contact with food by both the US FDA [7, 8] and the EU Plastics Regulation [9]. Other uses include biomedical applications [10], as ultrathin film of liquid lubricant in magnetic recording devices to reduce friction and deterioration due to the slider-disk contact [11–13]. The surface properties of PFPEs, including their tribology, are dependent on their viscosity, molecular structure, and degree of polymerization [1].

The different PFPE formulations in the market today have their backbone structures synthesized using manufacturer-specific processes based on a combination of techniques like anionic polymerization, hydrolysis, exhaustive fluorination, and photo-induced oxidation polymerization from raw materials such as 2,2,3-trifluoro-3-(trifluoromethyl)oxirane, 2,2,3,3-tetrafluorooxetane, tetrafluoroethene, and/or hexafluoropropene [2, 14], cf. Solvay-Solexis data sheet on Fomblin (http://www.solvay.com/en/markets-and-products/featured-products/Fomblin.html). Some commercially-available PFPE bases are known by their trade names such as Krytox, Fomblin, and Demnum [2]. The PFPE bases can have -CF_2_O-, −C_2_F_4_O-, −C_3_F_6_O-, or a random combination of these repeating units with molecular weights ranging from 500 to 15,000 Da [2]. The complexity in the chemical composition presents an analytical challenge in elucidating the structures of the individual species present in a PFPE formulation. In general, studying polymeric structures includes understanding the different features that contribute to their diversity. These features include: (1) molecular weight distribution; (2) nature of the repeating units; (3) presence of end groups, side chains, and other functionalities [15].

Mass spectrometry (MS), in recent years, has been widely used in the characterization of polymers. Soft ionization techniques like matrix-assisted laser desorption ionization (MALDI) and electrospray ionization (ESI) make possible performing MS analysis of large, intact molecules with minimum fragmentation [16–18]. MS is a promising tool that can be used to determine molecular weight distribution and molecular weight averages traditionally derived using size-exclusion chromatography (SEC). One advantage of MS over SEC is that MS can give additional information about absolute molecular weights, copolymerization, repeating units, and chemical structure of the individual molecule present in a given formulation.

High resolution mass spectrometry (HRMS) is a powerful tool that has been used to obtain more information regarding the chemical structure of polymers. For example, Fourier-transform ion cyclotron resonance (FT-ICR) MS has been widely used to characterize the end group of different polymers [19–21]. On the other hand, Orbitrap MS has been combined with a variety of innovative ionization techniques, including direct analysis in real time (DART) [22], desorption electrospray (DESI) [23], and atmospheric solids analysis probe [24]. Time-of-flight (TOF) is the most popular mass analyzer in polymer characterization, especially because it is easily interfaced with MALDI [16, 17]. Elucidation of polymer structures has been accomplished using tandem MS with TOF mass analyzers [18]. HRMS is particularly useful in studying polymer degradation [25, 26] and, in combination with SEC, in determining the molecular weight distribution of polymers [27, 28].

The next challenges in the mass spectrometry of polymers would include the development of ionization techniques to enable analyzing of hardly-ionizable types, for example, the PFAS-based polymers. So far, only the PFPEs were analyzed by MS [29, 30]. The development of a better mass spectral data processing tool is another challenge. This would be essential in the post-analysis of the mass spectra of highly complex and diverse polymer formulations like the PFPEs in the market today.

In recent years, the use of mass defects (MD) has become increasingly popular as a way of screening compounds in processing of high resolution mass spectra in non-target analyses. MD analysis has been successfully applied as a data mining tool in the identification of the different components of very complex mixtures like crude oil and extracts from biological matrices [31, 32].

The mass spectrum of complex polymer formulations can contain thousands of unique *m/z* peaks arising from different combinations of repeating units (or base units) and end groups. A way to reduce the number of data points is to adjust the mass scale in which the mass of a repeating unit is given a whole number value. For example, in the Kendrick mass scale, the mass of -CH_2_- unit is taken as 14.0000 u [33]. In this mass scale, compounds that have the same end group and are only varying in terms of the number of -CH_2_- units have equal mass defects. The plot of nominal mass versus Kendrick mass defect has been successfully used in the compositional discrimination of the components of poly(alkylene oxide) blends and of poly(ethylene oxide)/poly(propylene oxide) copolymers, and in profiling the end groups of poly (ε-caprolactone) [34]. PFPEs and other poly- and perfluorinated alkyl substances (PFAS), being dominated by the F nuclide (F has a negative mass defect), are characterized by negative mass defects (NMDs). NMDs can therefore be used as one of the initial criteria in screening these substances [35]. Roach and co-workers introduced the concept of higher-order mass defects as an extension of the “concept of Kendrick transformation to multiple bases” [36] . Higher-order mass defect analysis enables the elucidation not only of primary repeating units but also of other functional group differences. The first-order mass that is normalized relative to the first repeating unit (base), B_1_ (${M}_{B_1}^1(peak)$), can be calculated using Equation 1:

1 $$ {M}_{{\mathrm{B}}_1}^1(peak)=\frac{\mathrm{round}\ \left({\mathrm{M}}^0\left({\mathrm{B}}_1\right),\ 1\right)}{{\mathrm{M}}^0\left({\mathrm{B}}_1\right)}\ {\mathrm{M}}^0(peak) $$

where M^0^(B_1_) is the 0th-order mass of the repeating unit B_1_; and M^0^(*peak*) is the 0th-order mass of the ion. The first-order mass defect can be calculated using Equation 2:

2 $$ {\mathrm{M}\mathrm{D}}_{{\mathrm{B}}_1}^1(peak)={\mathrm{M}}_{{\mathrm{B}}_1}^1(peak)-\mathrm{round}\left({\mathrm{M}}_{{\mathrm{B}}_1}^1(peak)\right) $$

The second-order mass, which is normalized consecutively against the first and second repeating units B_1_ and B_2_, is calculated using Equation 3. Consequently the secon order mass defect can be obtained using Equation 4.

3 $$ {M}_{{\mathrm{B}}_1{\mathrm{B}}_2}^2(peak)=\frac{{\mathrm{M}\mathrm{D}}_{{\mathrm{B}}_1}^1\ \left(\mathrm{peak}\right)}{{\mathrm{M}}_{{\mathrm{B}}_1}^1\left({\mathrm{B}}_2\right)} $$

4 $$ M{D}_{{\mathrm{B}}_1{\mathrm{B}}_2}^2(peak)={M}_{{\mathrm{B}}_1{\mathrm{B}}_2}^2(peak) - \mathrm{ceiling}\left({M}_{{\mathrm{B}}_1{\mathrm{B}}_2}^2(peak),1\right) $$

In the cited work, calculation of up to the third-order mass defect enabled the data reduction and comparison of complex mass spectra of crude oil [36]. This can also be applied to other complex mixtures like copolymers with more than one kind of repeating units. The ceiling, round, or floor functions can be used depending on the nature of the mass defects being studied.

In this research work, the composition of a PFPE-based formulation was characterized by HRMS, specifically using ESI with quadrupole-time-of-flight (QqTOF) and Orbitrap mass analyzers. Mass spectral data interpretation was improved by employing first-, second-, and third-order mass defect analyses. Tandem MS up to the fourth-order was done on the most abundant ion to acquire information on the structural features of the ion including its end group. Similar ions were monitored in the fragmentation of other components after elution in C18 column. The elution order of the different components of the formulation in a C18 chromatographic column provided some complementary information regarding the repeating units, the formulation, and the influence of these features on the overall polarity of the polymeric species.

The information regarding chemical structure of polymers provides vital insights as to the properties these substances exhibit. This knowledge enables the manufacturers to fine-tune the production processes towards a specific application through quantitative structure–property relationship studies. Chemical data of polymers can also aid in studying the fate, behavior and degradability [37, 38] of these substances in the environment. This is particularly important for poly/perfluorinated polymers like the PFPE, as they can be precursors to highly persistent and/or toxic perfluorinated pollutants [39, 40].

## Materials and Methods

### Unknown PFPE-Based Formulation and Chemicals

The PFPE-based formulation was Solvera PT5045; Solvay Solexis, Bollate, Italy. For the experiments done at the University of Amsterdam, Institute for Biodiversity and Ecosystem Dynamics (UvA-IBED), the following reagents were used: methanol (ULC/MS grade, absolute) and formic acid (ULC/MS grade, 99%) purchased from Biosolve (Valkenswaard, The Netherlands); sub-boiled purified water prepared in-house. For the experiments done at the Hochschule Fresenius (HSF, Idstein, Germany), the following reagents were used: methanol and formic acid purchased from Carl Roth (Karlsruhe, Germany), and Milli-Q water prepared using a Milli-Q system with Simpak2 ion exchanger (Millipore, Milford, MA, USA).

### High Resolution MS

Two different mass spectrometers were used in this research study. The maXis 4G QTOF (QqTOF) (Bruker Daltonics, Bremen, Germany ) was used in the preliminary survey of the PFPE formulation. The working solution was prepared by diluting the PFPE-based formulation by a factor of 10,000 in methanol with 0.2% formic acid. The working solution was then introduced into the ESI source via a syringe pump at a 3 μL/min flow rate. The positive and negative mode TOF mass spectra were taken after instrumental parameter optimization in the range from 500 to 3000 u. The following were the optimized ESI parameter values: capillary, 5 kV; nebulizer gas, 0.4 bar; dry gas, 4.0 L/min; and dry temperature, 180°C. The mass analyzer settings were: funnel rf, 400 V_p-p_ (peak-to-peak voltage); multipole rf, 400; quadrupole ion energy, 4.0 eV; collision energy, 8.0 eV; collision rf, 3500 V_p-p_; transfer time, 75 μs; and pre-pulse storage, 35 μs. Mass calibration was performed using a polypropylene glycol standard provided by the instrument manufacturer.

Chromatographic separation was done using Shimadzu Nexera UPLC (Shimadzu, Duisburg, Germany) equipped with LC-30 AD pumps and IL-30 AC autosampler. The stationary phase was Phenomenex Luna C18 (II), 150 mm length, 3.0 mm internal diameter, 3 μm particle size and 100 Å pore size. Gradient elution was performed using 0.2% formic acid in 95:5 water:methanol (eluent A) and 0.2% formic acid in methanol (eluent B). Eluent ratio was varied from 40% eluent B to 100% eluent B in a total run time of 20 min: gradient was first increased to 80% eluent B at a rate of 20%/min; then, gradient was increased to 100% eluent B at a rate of 2%/min; the gradient was maintained at 100% eluent B for 11 min; the gradient was put back to 40% eluent B and was re-equilibrated. The mobile phase flow rate was maintained constant at 0.20 mL/min. The injection volume was 5 μL.

In another set of experiments, particularly to study the mass defects and the fragmentation of the polymer, the Orbitrap VelosPro, Hybrid Linear Ion Trap/Orbitrap MS (Thermo Scientific, Waltham, MA, USA) was used. The working solution was prepared by diluting the PFPE-based formulation by a factor of 1000 in methanol with 0.2% formic acid. The working solution was introduced into the MS via a syringe pump at 3 μL/min. The following were the optimized ESI parameter values: sheath gas flow rate, 30 (arbitrary unit, arb); aux gas flow rate, 20 (arb); spray voltage, 3.5 kV; and capillary temperature, 320°C. The lenses were tuned prior to use.

### MS Data Processing and Higher-Order Mass Defect Analysis

Data were processed using the accompanying software for each instrument. The Data Analysis 4.2 (Bruker Daltonics) and the Thermo Xcalibur 2.2 Qual browser (Thermo Scientific) were used for the QqTOF and Orbitrap data respectively. Additionally, the ChemCalc molecular formula finder [41] was used aside from the equivalent built-in chemical formula functions in the software.

The mass spectral data from the Orbitrap were extracted into a Microsoft Excel 2007 sheet and saved as comma delimited files. The raw Excel file was then processed by sorting the data according to decreasing relative intensity. The peaks with relative intensities less than 5% were not included in the higher-order mass defect calculations. The peaks were assumed to be singly-charged, thus *m/z* = m. The initial m values used were rounded-off to the nearest ten-thousandths.

The free software ‘R’ (The R Foundation for Statistical Computing, Vienna, Austria) was used in the higher-order mass defect calculations and in the generation of the mass defect plot. An R source code, ‘MassDef’, was developed in-house for this purpose. The ‘MassDef’ code makes use of several user-defined functions to generate different plots, e.g., molecular weight versus first-order mass defect. The plotting was made possible using the ‘ggplot2’ package [42]. The algorithm developed for the source code was based on the calculation of higher-order mass defects as described in detail in the work of Roach et al.[36].

### Tandem Mass Spectrometry

Two fragmentation modes in the Orbitrap Velos-Pro MS were used in the MS^n^ experiments: higher energy C-trap dissociation (HCD) and resonant collision-induced dissociation (termed ‘CID’ in the software).The ion with *m/z* 1176.999 was studied under different fragmentation parameters. The resulting fragment ions were then analyzed in the Orbitrap and in the linear ion trap. Initially, the ion with *m/z* 1176.999 was fragmented in the HCD cell at an increasing normalized energy of from 20% to 35%. The optimum normalized energy was found to be around 20%. Then, MS^n^ analysis up to the fourth-order was performed using stepwise CID starting from *m/z* 1176.999. The following precursor ions were fragmented using a normalized collision energy of 20%: *m/z* 1079, 1035, and 991.

The fragmentation of the other polymer moieties was also studied using HPLC-ESI-LIT-Orbitrap in the data-dependent acquisition (‘fifth-order double play’) mode.

Chromatography was carried out on an MZ Aqua C18 column (50 × 2.1 mm, 5 μm particle size; MZ Analysentechnik, Mainz, Germany) protected by a corresponding precolumn (10 × 2.1 mm). Eluents were A: H_2_O/MeOH (95/5; v/v) and B: H_2_O/MeOH (5/95; v/v), both containing 5 mM ammonium acetate. The components were separated by gradient elution starting at 50% A for 1 min followed by a linear decrease to 0% B within 10 min. After rinsing the column at 0% A for 5 min, the system was brought back to the starting conditions within 2 min and re-equilibrated at 50% A for 7 min. The flow rate was 200 μL/min and the injection volume was 10 μL.

Full scan spectra were recorded in the range of *m/z* 400–2000 in the Orbitrap at a nominal resolution setting of 60,000 (at *m/z* 400) and the five most intense ions within one scan were subsequently subjected to CID. These MS/MS spectra were recorded at normalized collision energy of 21.5% and stepped collision energy (three steps, 3%). Dynamic exclusion was activated (repeat count: 1; repeat duration: 30 s; exclusion list size: 300; exclusion duration: 30 s; exclusion by mass: 0.01) to prevent recording of MS/MS spectra of the same ions.

## Results and Discussion

### MS Analysis of PFPE-Based Formulation

A survey of the molecular weight distribution and the possible components of the PFPE-based formulation was initially done using ESI with Orbitrap and QqTOF mass analyzers. The mass errors after calibration (residuals) were all below 1 ppm. The QqTOF provides a mass resolution of up to 50,000. The mean value of 10 determinations for *m/z* 1176.9991 has an expanded uncertainty of ±0.0050 [95% confidence interval (CI)]. The expanded uncertainty is calculated from the standard error of the mean of n determinations multiplied by a coverage factor of 2 (95% CI). The Orbitrap, on the other hand, was calibrated as specified by the manufacturer. The mass errors after calibration (residuals) were all below 1 ppm. It has a mass resolution of up to 100,000 at *m/z* 400. The mean of 10 determinations has an expanded uncertainty of ±0.0005 (95% CI) for *m/z* 1176.9996.

**Figure 1:**
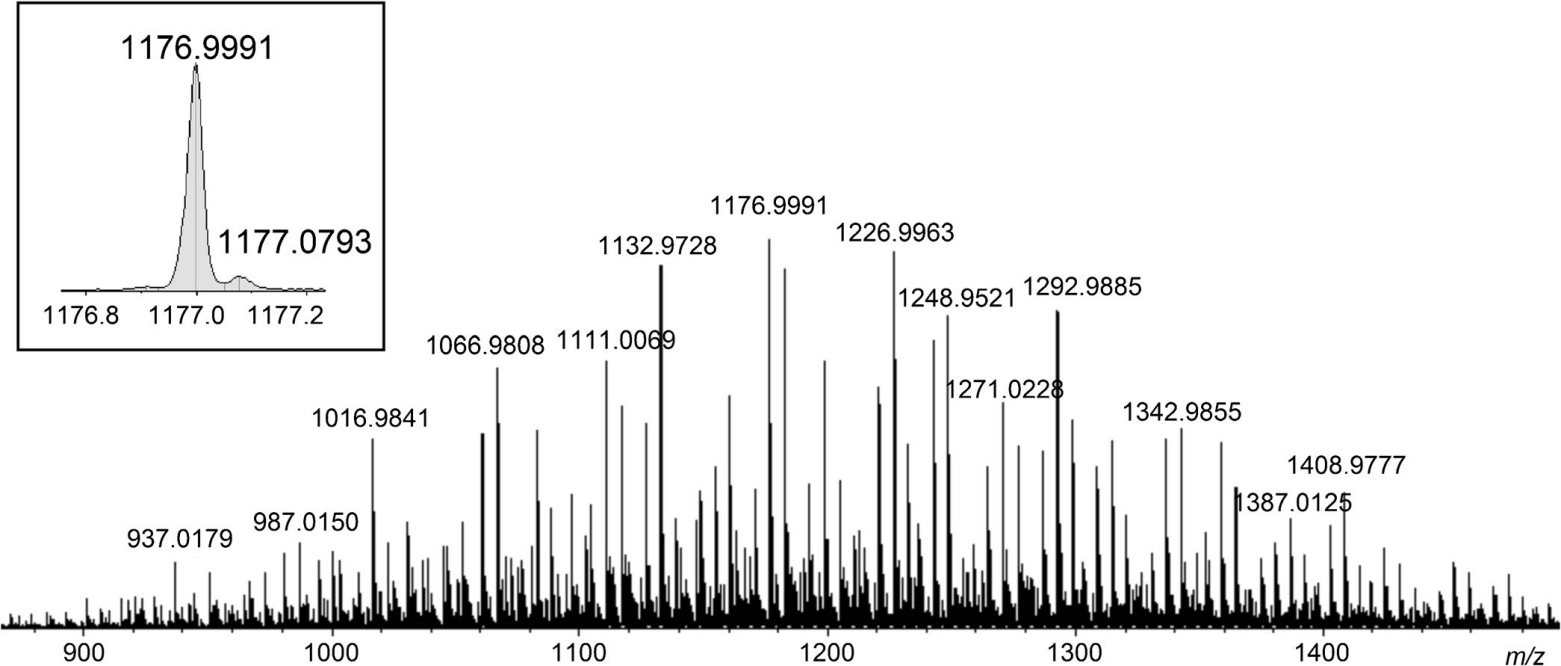
Positive-mode QqTOF mass spectrum of the PFPE-based formulation. Insert: magnified view of the peak with highest intensity

The generated average QqTOF positive-mode mass spectrum is shown in Figure 1. Most of the observed positive ions are in the *m/z* range between 900 and 1500. The base peak had an *m/z* of 1176.999 (Figure 1, insert). This *m/z* has an expanded uncertainty of ±0.020 (n = 10, CI = 95%). The nearby low-intensity peak with *m/z* of 1177.079 can easily be mistaken as an isotopolog. It will be shown, however, that the species with *m/z* of 1177.079 is not an isotopolog of *m/z* 1176.999 but a chemically distinct moiety because it has a different retention time in the HPLC. The generated positive ions are singly charged. Using the Data Analysis 4.2 software, the mass spectrum was screened for patterns of *m/z* differences. Patterns identified were consistent with -C_2_H_4_O- (44.026 u), −C_2_F_4_O- (115.989 u), −CF_2_- (49.997 u),and -CF_2_O- (65.992 u) moieties. These identified differences can be the possible repeating units of the multiblock copolymer.

The obtained positive-mode Orbitrap mass spectrum has a comparable mass distribution to that obtained using the QqTOF. The peak with highest intensity has an *m/z* of 1176.9994. This *m/z* has an expanded uncertainty of ±0.002 (n = 10, CI=95%).

ChemCalc Molecular Formula Finder [41] was used to predict the most likely chemical formula of the most intense ion in the QqTOF mass spectrum. Filter parameters were applied on the basis of prior observations regarding the possible repeating units and on the assumption that the molecules will not contain many other elements. Thus, it was preselected that the search will include the following: C: 0–50; H: 0–100; F: 0–100; O: 0–50, and P: 0–3. The ring and double bond equivalence (RDBE) was set to be between −2 and 2. The upper limit of the degree of unsaturation was set to a low value based on the information that the identified repeating units were saturated.

The most likely chemical formulae of the ion with *m/z* 1176.999 are [C_25_O_10_F_37_H_13_ + H]^+^ and [C_23_H_24_O_20_F_26_P_2_ + H]^+^ and the calculated differences of their corresponding exact masses to the obtained mass are 0.44 ppm and 0.68 ppm, respectively. The other *m/z* values can subsequently be assigned with the corresponding chemical formula on the basis of the difference pattern.

The most likely chemical formulae do not contain sodium. Unlike many polymers that form sodium adducts, the main ions observed for the PFPE formulation are protonated adducts. A hypothesis could be that the PFPE molecules in this study contain only a limited number of unhindered oxygen atoms that renders it unable to form sodium adducts. A molecular dynamic simulation study by Jonkers et al. suggests that the ethoxylate groups can wrap themselves around the sodium ion in such a way that the electron density of oxygen has optimum interaction with the cation [43]. In PFPE, the oxygen electron density can be hindered by the fluorine atoms. Therefore, formation of sodium adducts will only be possible when there is a substantial number of -C_2_H_4_O- units in a molecule.

### Analysis of Mass Defects

**Figure 2:**
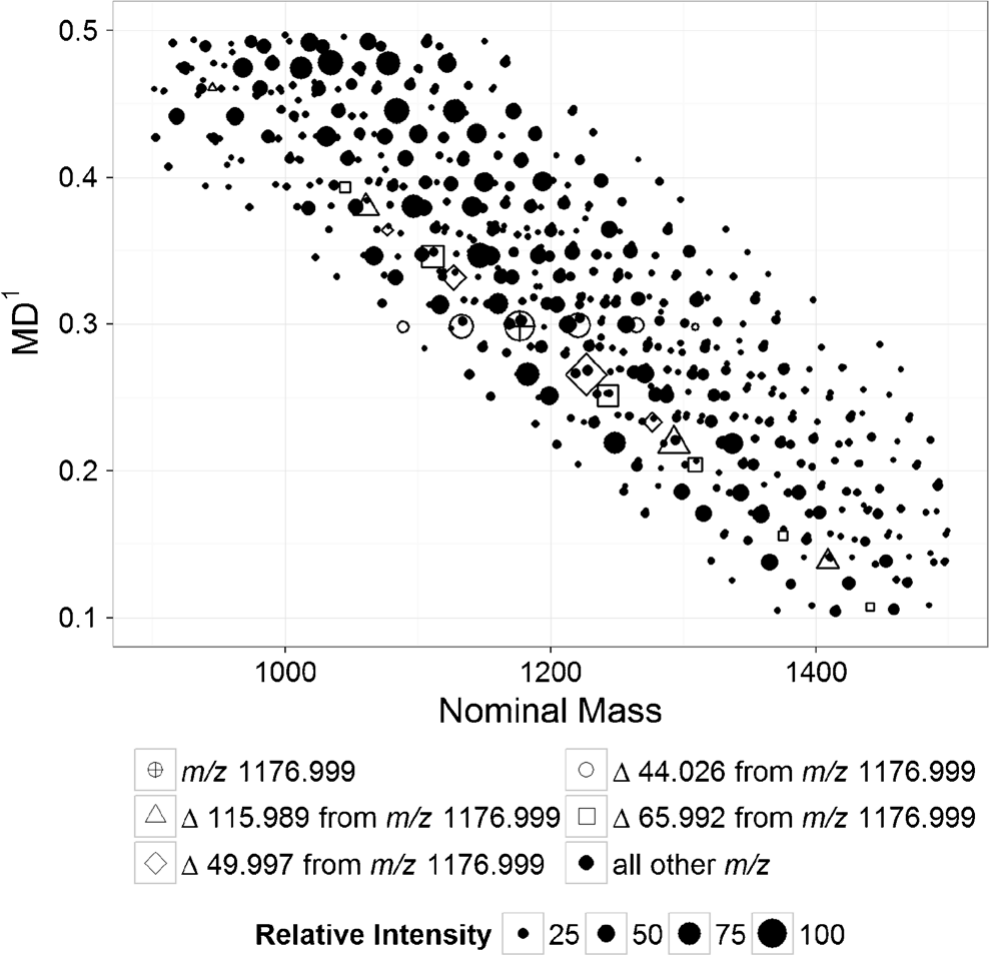
Plot of Molecular weight versus MD^1^ (generated using ‘MassDef’). The first-order mass transformation was done relative to the -C_2_H_4_O- repeating units. The values of the molecular weight used were acquired from the positive-mode ESI-Orbitrap mass spectral data with relative intensities greater than 5%

The positive-mode ESI-Orbitrap MS data of the PFPE-based formulation was used in the analysis of mass defects because the data is more precise. The first-order mass was calculated using Equation 1 with -C_2_H_4_O- as the repeating unit (B_1_). The detailed description of the calculation using the data in this study is added as Supplementary Information. Figure 2 shows the plot of the acquired molecular weights versus the first-order mass defect (MD^1^). The mass defect was calculated in absolute value to eliminate the negative sign. The “first-order” qualifier is used to denote the first cycle of mass defect calculation. The size of the points is proportional to the corresponding relative ion intensity. To visualize what happens during the mass defect calculation, *m/z* 1176.999 and the masses related to it by some repeating units were monitored. The *m/z* of the repeating units monitored included 115.989 (−C_2_F_4_O-); 44.026 (−C_2_H_4_O-), 65.992 (−CF_2_O-), and 49.997 (−CF_2_-) These *m/z* values are shown as different markers in Figure 2. It can be observed that all the *m/z* of 1176.999 ± n * 44.026 are aligned horizontally, indicating that they have equal MD^1^. This is consistent with the fact that the mass was scaled with respect to -C_2_H_4_O- unit. The molecules with equal MD^1^ values vary only in the number of the -C_2_H_4_O- units. All the other atoms in those molecules are exactly the same. The other *m/z* monitored, e.g., those varying by 115.989, 65.992, and 49.997 repeating units from 1176.999, follow a decreasing trend in MD^1^ with increasing molecular weight. The increase in the number of F and O nuclei in a molecule led to the overall decrease in MD^1^ since the atoms of these elements have negative mass defects.

**Figure 3:**
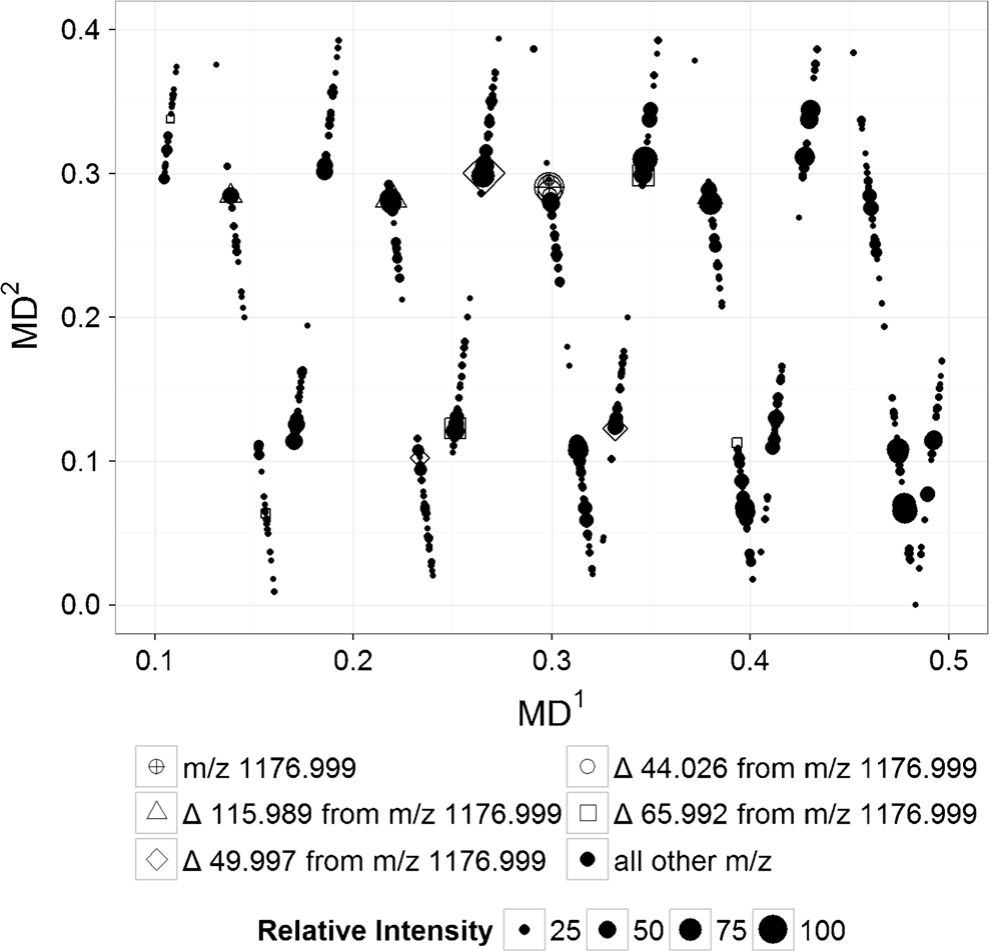
Plot of MD^1^ versus MD^2^ (generated using ‘MassDef’) from data acquired from the analysis of PFPE polymer by positive-mode LC-ESI-Orbitrap. The second-order mass transformation was done relative to the -C_2_F_4_O- repeating units

Another cycle of mass scaling and mass defect calculations can be employed [36] to detect other patterns present in a complex mixture. The second-order mass transformation was done with respect to the -C_2_F_4_O- repeating unit using Equation 3. The second-order mass defect (MD^2^) was then calculated. The plot of MD^1^ versus MD^2^ is shown in Figure 3. It can be observed that the points with equal MD^1^ in Figure 2 clustered together into just a single point in Figure 3. All points with equal MD^1^ will always have equal MD^2^ because they have exactly the same atoms and are only varying in the number of -C_2_H_4_O- units. After the second-order transformation, all the points with *m/z* varying by 115.989 from 1176.999 will have equal MD^2^. These molecules vary in the number of both the -C_2_H_4_O- and -C_2_F_4_O- units and all the other atoms in those molecules are exactly the same.

**Figure 4:**
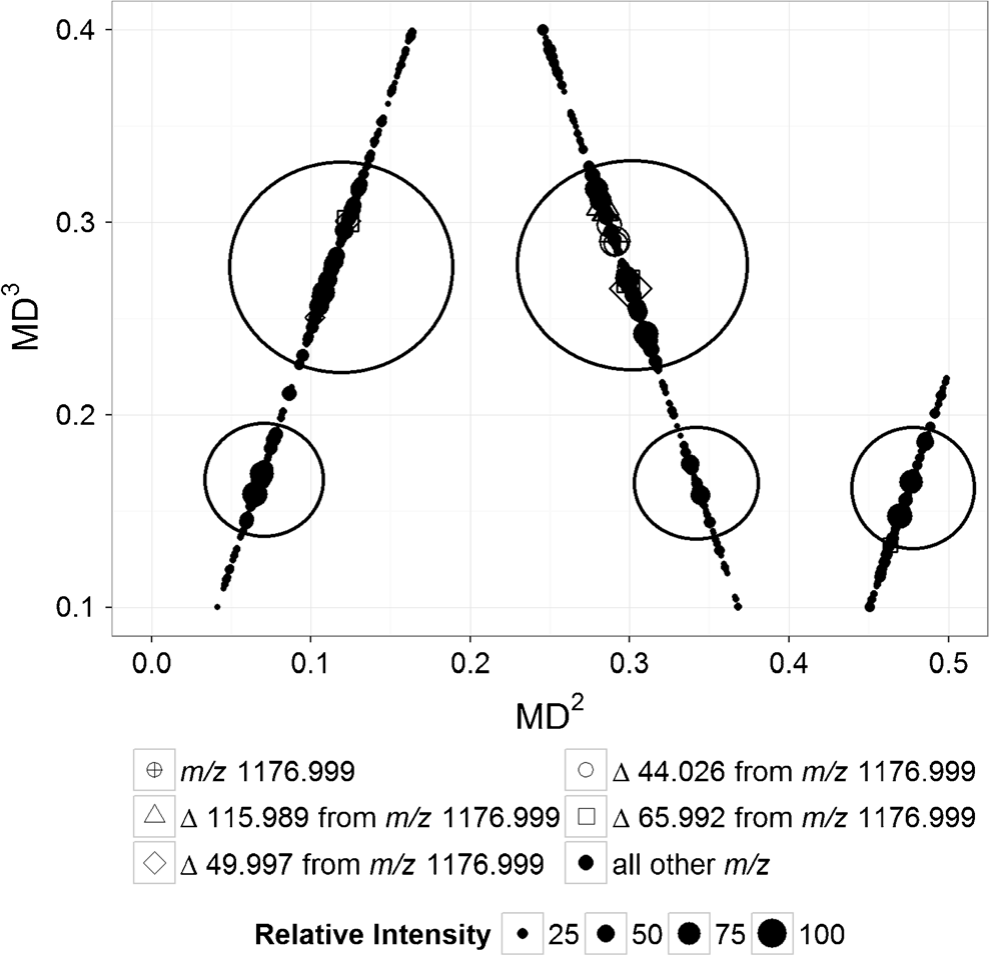
Plot of MD^2^ versus MD^3^ (generated using the ‘MassDef’) from data acquired from the analysis of PFPE polymer by positive-mode LC-ESI-Orbitrap. The third-order mass transformation was done relative to the CF_2_O repeating units. The circled regions represent clusters of points with mass defects assumed to be not significantly different from each other

The third-order mass transformation was done relative to -CF_2_O- unit and the plot of MD^2^ versus the third-order mass defect (MD^3^) is shown in Figure 4. In this plot, five clusters of points can be observed: two large clusters and three small clusters. The large clusters are in the same range of MD^3^ (0.23–0.33). Likewise, the small clusters are all in another MD^3^ range (0.14–0.20). Ideally, it is expected that all the *m/z* that vary only in the number of -CF_2_O- from 1176.999 units will have equal MD^3^. In Figure 4, the points are in a certain range instead of being in a single horizontal line that denotes equal MD^3^. It is shown in the sample calculations presented in Supplementary Information 1 that the variations in the values of MD increase with the order of transformation. The values of MD^3^ have a variation of ±0.04 (2*SD). The precision of MS data is a limiting factor on how far one can perform higher-order mass transformation for mass defect calculations. The spreading of the MD^3^ values within a certain range, therefore, can be attributed to enlarged effect of noise and the low value of the mass defect of the -CF_2_O- repeating unit.

It is shown in this section that higher-order mass defect is a powerful tool that can be used not just in screening the perfluorinated polymers but also in surveying the different repeating units and the extent of their variability in a given polymer mixture.

### Tandem Mass Spectrometry

**Scheme 1:**
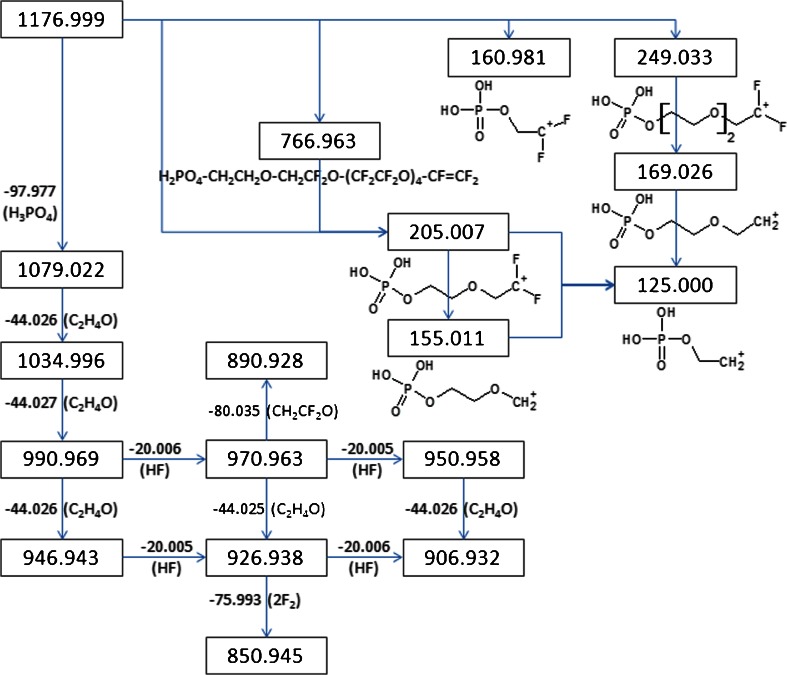
Orbitrap MS^n^ fragmentation of the ion with *m/z* 1176.999

So far, the data from HRMS including the mass defect analysis is not sufficient to predict the general structure of the polymer. MS^n^ was done to elucidate the structural features of the molecules present in the PFPE formulation. Both the LIT and Orbitrap mass analyzers were used in this experiment. The number of significant figures of the reported *m/z* reflects the accuracy of the mass analyzer used. The ion with *m/z* 1176.999 has the most intense signal in the mass spectrum; thus, it was chosen for the extended MS^n^ study. The MS^n^ fragmentation of the ion with *m/z* 1176.999 is summarized in Scheme 1. The supporting mass spectra are included in the Supplementary Information. The high-MW principal product ions of *m/z* 1176.999 result from the loss of H_3_PO_4_ (97.977 *m/z*) and the successive losses of C_2_H_4_O (44.026 *m/z*). The other high-MW product ions result from the loss of HF (20.006 *m/z*) of the main fragments. On the other hand, most of the low mass product ions contain phosphate functional groups. MS^3^ was performed on the major product ions of *m/z* 1176.999: *m/z* 1079.022, 1034.996, and 990.969. The mass spectra obtained showed successive losses of 44.026 *m/z* as was previously observed. This also confirms the linear nature of the polymer molecule. The product ion of *m/z* 1177.0 with *m/z* of 991.0 was further fragmented in the CID. The main product ions have *m/z* of 970.963 and 946.943. These correspond to the losses of HF and C_2_H_4_O, respectively. MS^4^ was done on the product ion with *m/z* 971.0 following the transition: 1177.0 → 991.0 → 971.0. The product ions have *m/z* values of 951.0 and 926.9. These ions correspond to the losses of HF and C_2_H_4_O, respectively. The product ion with *m/z* 906.9 results from the successive losses of HF and C_2_H_4_O. Interestingly, not all the ions produced in the fragmentation of *m/z* 991.0 are also generated in the fragmentation of *m/z* 970.9, particularly *m/z* 946.9. The ions with *m/z* of 970.9 and 946.9 can be generated from either different positional isomers or from the same but asymmetric polymer molecule. The product ion with *m/z* 890.9 was also observed. This is the result of the loss of C_2_H_2_F_2_O from *m/z* 970.9.

The mass spectra derived from data-dependent acquisition coupled to chromatographic separation reveal that the patterns of fragmentation observed from ion 1176.999 *m/z* were similar to those of the other ions (see table in the Supplementary Information). The loss of 97.977 *m/z* is indicative of the presence of a phosphate group in the PFPE molecules. The series of 44.026 *m/z* losses points to the cleaving off of C_2_H_4_O.

### Reversed-Phase HPLC

**Figure 5:**
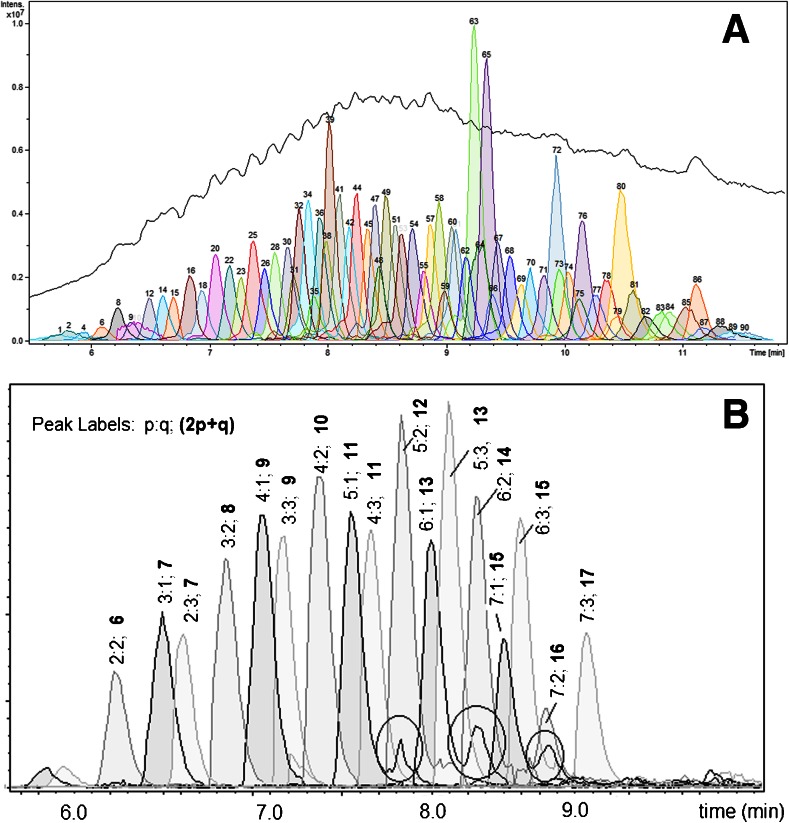
(**a**) Base peak chromatogram of the PFPE formulation and the Dissect chromatogram traces; (**b**) chromatograms of selected ions that correspond to the chemical formula: (OH)_2_OPO-(C_2_H_4_O)_n_-(C_2_H_2_F_2_O)_o_-(C_2_F_4_O)_p_-(CF_2_O)_q_-PO(OH)_2_. The data were obtained from the positive-mode LC-QqTOF

Reversed-phase HPLC was used to separate the different components of the formulation based on polarity. Shown in Figure 5a is the base peak chromatogram of PFPE formulation obtained with the QTOF-MS. Superimposed in the same figure are the ‘Dissect’ chromatogram traces resulting from the deconvolution process. The dissect algorithm of the Data Analysis software combines all ions from molecules with similar chromatographic elution profile into one chromatographic trace. At a certain time window, if two or more ions have the same retention times and peak widths, and if they have similar shapes, they are combined as one chromatographic trace. In Figure 5a, the 90 software-derived chromatographic traces represent 90 groups of compounds with different elution properties in a C18 stationary phase. An inspection of the mass spectrum of a chromatogram trace reveals that the molecules with the same elution properties showed a pattern of 44.026 *m/z* mass differences. This pattern can be attributed to -C_2_H_4_O- repeating units. This implies that species varying only in the number of -C_2_H_4_O- repeating units are not separated by this particular C18 HPLC column, which is consistent with reverse phase chromatographic behavior observed, for example, alkylphenolethoxylates [44], although separation of such PFPE-ethoxylate oligomers recently has been obtained on a C18 UHPLC column [5].

Based on the results discussed in the preceding sections, a major component of the polymer formulation will have the general formula (1):

1 $$ {\left(\mathrm{O}\mathrm{H}\right)}_2\mathrm{O}\mathrm{P}\mathrm{O}-{\left({\mathrm{C}}_2{\mathrm{H}}_4\mathrm{O}\right)}_{\mathrm{n}}-{\left({\mathrm{C}}_2{\mathrm{H}}_2{\mathrm{F}}_2\mathrm{O}\right)}_{\mathrm{o}}-{\left({\mathrm{C}}_2{\mathrm{F}}_4\mathrm{O}\right)}_{\mathrm{p}}-{\left({\mathrm{C}\mathrm{F}}_2\mathrm{O}\right)}_{\mathrm{q}}-\mathrm{P}\mathrm{O}{\left(\mathrm{O}\mathrm{H}\right)}_2 $$

The elution order of selected species having this general chemical formula was monitored as shown in Figure 5b. In general, the retention in the C18 column of the polymer molecule is proportional to the total number of -CF_2_- units given by the formula 2p+q. If the number of CF_2_ units are equal, the higher the sum of C_2_F_4_O and CF_2_O (p+q), the longer is the retention time.

Some shoulders were observed in the chromatogram as shown in Figure 5b. These shoulders are in reality a set of masses different from the ones being monitored. The *m/z* values that caused the shoulders were 0.095 different from the *m/z* values being monitored. For example, the *m/z* values of 1248.9619 and 1249.0571 have chemical formulas C_23_H_21_F_30_O_20_P_2_ (n = 3; p:q = 5:3) and C_27_H_33_F_26_O_21_P_2_ (n = 6; p:q = 5:1), respectively. These two will have different retention times. The software settings to generate the chromatograms were not discriminating enough between these two sets of masses with a difference of less than 0.1 *m/z*.

Figure 5b also shows the molecular diversity of the PFPE formulation. The numbers of -C_2_F_4_O- and -CF_2_O- repeating units in a molecule range from 2 to 7, and 2 to 3, respectively. The total number of -CF_2_- per molecule ranges from 6 to 17.

### Comparison of the Derived Chemical Structure to the Published Technical Data

The chemical structure is not given on the technical datasheet for the PT 5045, produced by Solvay Solexis. They do, however, mention that it is a perfluoropolyether-phosphate derivative, used for paper and board. In a 2013 patent application from the same company and where PT 5045 is a raw material, the average structure of the polymer is given as (2):

2 $$ \begin{array}{l}{\left(\mathrm{H}\mathrm{O}\right)}_2\mathrm{O}\mathrm{P}\mathrm{O}-{\left[{\left({\mathrm{CH}}_2{\mathrm{CH}}_2\mathrm{O}\right)}_{\mathrm{n}}-{\mathrm{CH}}_2-{\mathrm{R}}_{\mathrm{F}}-{\mathrm{CH}}_2-{\left({\mathrm{OCH}}_2{\mathrm{CH}}_2\right)}_{\mathrm{n}}-\mathrm{OP}\left(\mathrm{O}\mathrm{H}\right)\right]}_{0.1}{\left({\mathrm{CH}}_2{\mathrm{CH}}_2\mathrm{O}\right)}_{\mathrm{n}}-{\mathrm{CH}}_2-{\mathrm{R}}_{\mathrm{F}}-{\mathrm{CH}}_2-{\left({\mathrm{OCH}}_2{\mathrm{CH}}_2\right)}_{\mathrm{n}}-\mathrm{OP}{\left(\mathrm{O}\mathrm{H}\right)}_2\hfill \\ {}{\mathrm{R}}_{\mathrm{F}} = -{\mathrm{CF}}_2\mathrm{O}-{\left({\mathrm{CF}}_2{\mathrm{CF}}_2\mathrm{O}\right)}_{\mathrm{p}}-{\left({\mathrm{CF}}_2\mathrm{O}\right)}_{\mathrm{q}}-{\mathrm{CF}}_2-\hfill \end{array} $$

The patent specifies that p:q is between 2 and 3 and that n is 1.8 [45]. The structure suggests that a molecule can contain two or three P atoms. Ninety percent of the molecules contain only two P atoms that are at both ends of the molecules as phosphate esters.

The results of the present study suggest a structure similar to the one proposed by Solvay Solexis for PT5045, although the ratio p:q is more variable and ranges from 1:3 to 7:1 as shown in Figure 5b. Also, the distribution of n is not always symmetric. Positional isomers can exist and the number of -CH_2_CH_2_O- repeating units on both ends of the molecule beside the phosphate groups adds up to four as can be deduced from the plot of MW versus MD^1^ (Figure 2).

### Chemical Structure and Physico-Chemical Properties of PFPE-Based Formulations

In the past years, fluorinated polymeric coatings have become increasingly popular as a way to decrease the potential migration from food packaging in contrast to smaller PFASs, for example the fluorotelomer alcohols (FTOHs). First, the polymer is coated only at the surface, and second, the polymer is cross-bound to itself so it is less amenable to migrate. Engineering the endgroups and the number of each monomer unit in a PFPE molecule is a way to adjust the solubility in certain solvents and to enable their binding to a variety of polar to nonpolar surfaces. The presence of ether groups makes the polymer more water soluble by the ability of the oxygen lone pairs to donate electrons for hydrogen bonding. Water solubility can be further enhanced by the addition of an ionic terminal endgroup, called the polar head of the surfactant, which typically could be phosphates, carboxylates, or quaternary amines.

As surfactants, PFPEs may elicit significant physicochemical differences depending on the hydrocarbon segments (blocks) they contain. The hydrocarbon block enables them to make hydrophobic bonds to hydrocarbon surfaces. This increases the abrasion strength of other layers, such as printing inks, lacquers, or glue, which are to be tied onto the PFPE layer at the surface. On the other hand, perfluorinated ethers without the hydrocarbon block would repel the hydrocarbon chemicals. If these PFPEs are to be used in food packaging, the size of the hydrocarbon block must be controlled because of the increased risk to humans upon intake brought about by increased binding to lipids [10].

Currently, PFPE-based polymers are considered alternatives to persistent long-chain perfluoroalkyl carboxylic acids and sulfonates and their precursors. However, several questions arise regarding their degradability, persistence, and toxicity [46, 47]. These polymers can most likely be also precursors to persistent and slowly degrading compounds. For, example, Galden, a PFPE with -OCF_2_(CF_3_)CF- repeating units, was shown to degrade very slowly at atmospheric conditions [46, 48]. These data gaps can only be addressed properly beginning with accurate information regarding the chemical structures of the polymers derived from mass spectrometry studies. Lack of information of specific chemical substances, including technical blends, cannot be assessed by in-silico methods, such as quantitative structure property relations (QSPR) and quantitative structure activity relations (QSAR), which require knowledge of the specific molecular structure [49]. For the same reason technical mixtures are also often omitted from evaluation of toxicity by in-vitro and in-vivo studies (Nordic Report on PFAS 2013). This is of concern as it means that whole groups of chemicals by-pass critical evaluation. Introduction of tools to facilitate the identification and quantification of mixtures of chemicals is therefore highly needed, and in this respect this research paper provides a HRMS-based approach to the characterization of not only PFPE-based formulations but also other fluorinated and nonfluorinated polymers. Furthermore, the use of higher-order mass defects is of interest to other fields studying blends of chemicals, such as fracking liquids [50], petroleum, cosmetics, and more. In the future, these researches will be facilitated if mass defect calculations and plots will be included in the software updates of various MS products for HRMS screening.

## Conclusion

Industrial chemicals often come as mixtures of substances, as in the case of petroleum [50], detergents [51], or as polymers used in food contact materials. The complexity of the mixtures makes it time-consuming and challenging to identify and quantify the individual substances in the blends and in real samples. In this study, we demonstrated how the use of the higher-order mass defects helped reduce the complexity of the mass spectral data of a PFPE-based formulation by identifying the most likely repeating units that includes: −C_2_H_4_O-, -C_2_F_4_O-, and -CF_2_O-. Tandem MS was used to identify the end groups and the possible distribution of the repeating units. Reversed phase HPLC enabled the separation of the homologous series of individual polymer molecules on the basis of increasing numbers of the nonpolar repeating units. The structure was consistent with the structure supplied in the manufacturer’s technical data sheet, albeit containing more information on the individual polymer molecules and not just average data. The analytical approach presented in this research paper can be applied to other polymers as well. MS data as well as the mass defect graphs can be useful tools that can provide essential starting information. Eventually, the data gaps that hamper the assessment of the risks that these complex mixtures pose to the environment can be narrowed down.

## Electronic supplementary material

ESM 1 (DOCX 44 kb)
