# Supplementary material for: High Resolution Mass Spectrometry of Polyfluorinated Polyether-Based Formulation
Source: J Am Soc Mass Spectrom. 2015 Oct 30;27:309–18. doi: 10.1007/s13361-015-1269-9 (PMC4723628; doi:10.1007/s13361-015-1269-9)
Supplement: Supplementary file 1 — (DOCX 44 kb) [file 13361_2015_1269_MOESM1_ESM.docx]

High Resolution Mass Spectrometry of Polyfluorinated Polyether – based Formulation

Ian Ken Dimzon, Xenia Trier, Tobias Frömel, Rick Helmus,

Thomas P. Knepper and Pim de Voogt

Supplementary Information

**Supplementary Information 1**

Higher-order mass defect calculation

**SI 1a** Higher-order mass transformation of selected accurate masses from the mass spectrum of PFPE formulation.

| Accurate Mass, M^0^ (1) | Relative Intensity (2) | Relation to m/z 1176.9994 | | | 1st Order Transformation | | | 2nd Order Transformation | | | 3rd Order Transformation | | |
| --- | --- | --- | --- | --- | --- | --- | --- | --- | --- | --- | --- | --- | --- |
|  |  | -C_2_H_4_O- (3) | -C_2_F_4_O- (4) | -CF_2_O- (5) | M^1^ (6) | round(M^1^,0) (7) | MD^1^ (8) | M^2^ (9) | round(M^2^,0) (10) | MD^2^ (11) | M^3^ (12) | round (M^3^, 0) (13) | MD^3^ (14) |
| 1176.9994 | 100.0 |  |  |  | 1176.2990 | 1176 | 0.2990 | 3.7130 | 4 | 0.2870 | 0.7018 | 1 | 0.2982 |
| 1088.9465 | 79.5 | -2 |  |  | 1088.2985 | 1088 | 0.2985 | 3.7068 | 4 | 0.2932 | 0.7170 | 1 | 0.2830 |
| 1132.9731 | 78.4 | -1 |  |  | 1132.2989 | 1132 | 0.2989 | 3.7117 | 4 | 0.2883 | 0.7048 | 1 | 0.2952 |
| 1221.0259 | 78.0 | +1 |  |  | 1220.2993 | 1220 | 0.2993 | 3.7167 | 4 | 0.2833 | 0.6927 | 1 | 0.3073 |
| 1265.0523 | 77.9 | +2 |  |  | 1264.2995 | 1264 | 0.2995 | 3.7192 | 4 | 0.2808 | 0.6866 | 1 | 0.3134 |
| 1309.0776 | 76.8 | +3 |  |  | 1308.2986 | 1308 | 0.2986 | 3.7080 | 4 | 0.2920 | 0.7140 | 1 | 0.2860 |
| 945.0236 | 95.4 |  | -2 |  | 944.4612 | 944 | 0.4612 | 5.7280 | 6 | 0.2720 | 0.6651 | 1 | 0.3349 |
| 1061.0114 | 87.0 |  | -1 |  | 1060.3800 | 1060 | 0.3800 | 4.7192 | 5 | 0.2808 | 0.6865 | 1 | 0.3135 |
| 1292.9885 | 85.8 |  | +1 |  | 1292.2190 | 1292 | 0.2190 | 2.7203 | 3 | 0.2797 | 0.6837 | 1 | 0.3163 |
| 1408.9769 | 85.2 |  | +2 |  | 1408.1384 | 1408 | 0.1384 | 1.7190 | 2 | 0.2810 | 0.6869 | 1 | 0.3131 |
| 1045.0156 | 75.6 |  |  | -2 | 1044.3937 | 1044 | 0.3937 | 4.8896 | 5 | 0.1104 | 0.2699 | 0 | 0.2699 |
| 1111.0076 | 74.8 |  |  | -1 | 1110.3464 | 1110 | 0.3464 | 4.3025 | 4 | 0.3025 | 0.7397 | 1 | 0.2603 |
| 1242.9915 | 72.6 |  |  | +1 | 1242.2518 | 1242 | 0.2518 | 3.1271 | 3 | 0.1271 | 0.3108 | 0 | 0.3108 |
| 1308.9835 | 71.2 |  |  | +2 | 1308.2045 | 1308 | 0.2045 | 2.5400 | 2 | 0.5400 | 1.3204 | 1 | 0.3204 |
| **Standard deviation of shaded MD** | | | | | | | **0.008** |  | | **0.012** |  | | **0.020** |

**SI 1b** Higher-order mass transformation of the selected exact masses of the repeating unit

| Repeating Units | Order | Exact Mass | M^1^ | round(M^1^,0) | MD^1^ | M^2^ | round(M^2^,0) | MD^2^ | M^3^ | round(M^3^,0) | MD^3^ |
| --- | --- | --- | --- | --- | --- | --- | --- | --- | --- | --- | --- |
| -C_2_H_4_O- | 1 | 44.0262 | 44.0000 | 44 | 0.00000 | 0.0000 | 0 | 0.0000 | 0 | 0 | 0.0000 |
| -C_2_F_4_O- | 2 | 115.9885 | 115.9195 | 116 | 0.08052 | 1.0001 | 1 | 0.0001 | 0.0001 | 0 | 0.0001 |
| -CF_2_O- | 3 | 65.9917 | 65.9524 | 66 | 0.04757 | 0.5910 | 1 | 0.4090 | 1.0001 | 1 | 0.0001 |

Steps in the calculation of higher-order mass defects used in this study and the generation of the different MD graphs.

1. Accurate masses and their corresponding relative intensities were extracted from the mass spectrum of PFPE formulation (Columns 1 and 2 in SI1a).

2. It was decided that the transformations will be in the following order of the bases (repeating units): ‑C_2_H_4_O‑, ‑C_2_F_4_O‑, and ‑CF_2_O‑.

3. First order transformation (based on the ‑C_2_H_4_O‑ repeating units): M^0^ was multiplied by the ratio 44/44.0262 to obtain M^1^ (See SI1b). The nominal M^1^ was obtained by rounding off M^1^ to the nearest ones.

MD^1^ = M^1^ - nominal M^1^

4. Second order transformation (based on the ‑C_2_F_4_O‑ repeating units): M^2^ was calculated by dividing MD^1^ by the MD^1^ of ‑C_2_F_4_O‑ (0.08052). The nominal M^2^ was obtained by rounding off M^2^ to the nearest ones.

MD^2^ = M^2^ - nominal M^2^

5. Third order transformation (based on the ‑CF_2_O‑ repeating units): M^3^ was calculated by dividing MD^2^ by the MD^2^ of ‑CF_2_O‑ (0.4090). The nominal M^3^ was obtained by rounding off M^3^ to the nearest ones.

MD^3^ = M^3^ - nominal M^3^

6. Different graphs can be generated using SI1a, for example, nominal M^0^ vs MD^1^; MD^1^ vs MD^2^; etc. The relative intensities can be added in the graph as a third dimension (e.g. size of the points).

**Supplementary Information 2**

Sample MS^n^ mass spectra of *m/z* 1176.999.

**SI 2a** Fragmentation of *m/z* 1176.9 as precursor ion in the HCD at a normalized collision energy of 25%; recorded in the FTMS mode

**SI 2b** MS^3^ with the following precursor ions: 1176.9 🡪 1079.0 *m/z* in the CID at a normalized collision energies of 23% and 20% respectively; recorded in the FTMS mode

**SI 2c** MS^4^ with the following precursor ions: 1176.9 🡪 990.9 🡪 970.9 *m/z* in the CID at a normalized collision energies of 23%, 20% and 20% respectively; recorded in the ITMS mode

**Supplementary Information 3**

Summary of fragmentation of some major ions selected by data-dependent acquisition

| Precursor Ion (*m/z*)  (1) | Relation to *m/z* 1177 | | | | Observed Fragment Ions (*m/z*) | | |
| --- | --- | --- | --- | --- | --- | --- | --- |
|  | -C_2_H_4_O-  (2) | -C_2_F_4_O-  (3) | -CF_2_-  (4) | difference from *m/z* 1177 (5) | Neutral Loss of 18 *m/z* (6) | Neutral Loss of 98 *m/z* (7) | Series of 44 *m/z* losses starting from the *m/z* in column (7)  (8) |
| 945 |  | -2 |  | -232 | 927 | 847 | 803; 759; 715 |
| 1017 | -1 | -1 |  | -160 | 999 | 919 | 875; 831; 787 |
| 1061 |  | -1 |  | -116 | 1043 | 963 | 919; 875; 831; |
| 1105 | +1 | -1 |  | -72 | 1087 | 1007 | 963; 919; 875; 831 |
| 1111 |  | -1 | +1 | -66 | 1093 | 1013 | 969; 925; 881 |
| 1127 |  |  | -1 | -50 | 1109 | 1029 | - |
| 1133 | -1 |  |  | -44 | 1115 | 1035 | 991; 947 |
| 1155 | +1 | -1 | +1 | -22 | - | 1057 | 1013; 969; 925 |
| **1177** |  |  |  | **0** | **1159** | **1079** | **1035; 991; 947** |
| 1227 |  |  | +1 | 50 | - | 1129 | - |
